# Salivary free aldosterone awakening response and cardiovascular risk in men with CHD, hypertension, and healthy controls

**DOI:** 10.3389/fendo.2025.1655896

**Published:** 2026-01-05

**Authors:** Angelina Gideon, Roland von Känel, Cathy Degroote, Livia Thomas, Claudia Zuccarella-Hackl, Roland Wiest, Petra H. Wirtz

**Affiliations:** 1 Biological Work and Health Psychology, University of Konstanz, Konstanz, Germany; 2 Department of Consultation-Liaison Psychiatry and Psychosomatic Medicine, University Hospital Zurich, University of Zurich, Zurich, Switzerland; 3 Support Center of Advanced Neuroimaging, Institute of Diagnostic and Interventional Neuroradiology, University Hospital Bern, University of Bern, Bern, Switzerland; 4 Centre for the Advanced Study of Collective Behaviour, University of Konstanz, Konstanz, Germany

**Keywords:** aldosterone awakening response, biologically active aldosterone, coronary heart disease, essential hypertension, hypertension, salivary free aldosterone

## Abstract

**Background:**

The mineralocorticoid hormone aldosterone plays a key role in blood pressure regulation and the development of cardiovascular disease. However, the biological mechanisms that underly prospective associations with negative health outcomes are not fully understood. We compared the awakening response of biologically active salivary aldosterone (AldAR) between male participants with coronary heart disease (CHD), essential hypertension (EHT), and normotension (NT) and additionally examined prospective associations with biological CHD risk factors.

**Methods:**

At baseline, 60 CHD patients, 40 EHT and 44 NT repeatedly assessed the AldAR over two consecutive days. In 97 participants, prospective CHD risk was assessed by changes from baseline to follow-up 2.95 ± 0.07 (*SEM*) years later in the pro-inflammatory measures interleukin (IL)-6, tumor necrosis factor-alpha (TNF-α), and acute phase protein C-reactive protein (CRP), as well as blood lipids in terms of total cholesterol (tChol), high-density lipoprotein (HDL), and low-density lipoprotein (LDL) cholesterol. Potential confounding variables were controlled.

**Results:**

Whereas NT showed the regular AldAR, EHT had higher overall aldosterone levels (*p*’s < .010, with confounders: *p*’s < .065) but a flattened AldAR (*p* = .89). Moreover, CHD patients showed the regular AldAR but on a lower level as compared to NT (*p* = .002, with confounders: *p* = .075). Greater AldAR area under the curve independently predicted greater increases in overall inflammatory (*p*’s ≤ .032) and lipid CHD risk markers (*p*’s ≤ .089). Significantly greater increases were found for LDL cholesterol (ß = .24, *p* = .037; with confounders: *p*’s ≤ .067), and tChol (ß = .26, *p* = .032; with confounders: *p*’s ≤ .064). A trend for an association was found for IL-6 (ß = .22, *p* = .068; with confounders: *p*’s ≤ .079).

**Conclusions:**

We found evidence for altered AldAR in CHD and EHT with higher AldAR predicting higher CHD risk, particularly in relation to systemic inflammation and dyslipidemia. Elevated AldAR levels may contribute to the pathogenesis of atherosclerotic cardiovascular disease.

## 1 Introduction

Coronary heart disease (CHD) with atherosclerosis as the underlying mechanism is the leading cause of death in Western countries (1). One of the primary risk factors for CHD is hypertension (HT), a condition characterized by persistently elevated blood pressure (BP) that affects more than 30% of adults worldwide. The vast majority of HT patients is diagnosed with essential hypertension (EHT) with the cause for their condition being unclear (2).

A key factor in the regulation of BP is the mineralocorticoid hormone aldosterone that regulates extracellular volume and BP by balancing sodium and potassium (3). Released from the zona glomerulosa of the adrenal glands, aldosterone is primarily regulated by the renin-angiotensin-aldosterone system (RAAS), adrenocorticotropic hormone (ACTH), and potassium levels (3).

Aldosterone measurement from blood provides an assessment of both bound and unbound aldosterone (total aldosterone). Most of the aldosterone in an organism is bound to proteins, but around 35–40% remains unbound and biologically active and thus capable of exerting its biological effects (4). Recent studies have demonstrated that aldosterone can be reliably measured in saliva (5, 6) reflecting approximately 80% of the biologically active free aldosterone found in plasma (7). Salivary biologically active aldosterone levels strongly correlate with total plasma aldosterone (6) and are independent of saliva flow (5). Advantages of saliva sampling include its non-invasive nature, the ability to store samples at room temperature, and the convenience of self-administered, repeated collection in natural settings. Aldosterone follows a circadian pattern, with highest levels occurring in the first hour after awakening (aldosterone awakening response, AldAR) (8, 9) that gradually decrease throughout the day, with lowest concentrations in the evening (7, 8). Recently, Upton et al. (10) provided high-resolution 24-hour profiles of adrenal steroids, including aldosterone, using ambulatory microdialysis sampling combined with LC-MS/MS, demonstrating complex circadian rhythmicity in real-world conditions.

There is evidence for alterations in circadian aldosterone in HT. However, four studies that compared *circadian total plasma aldosterone* rhythms between HT patients and NT controls, using repeated daytime measurements every two (11), four (12, 13), and six hours (14) did not observe overall significant differences between groups. In contrast, studies assessing biologically active *circadian aldosterone from saliva* are few and found higher circadian aldosterone levels in patients with EHT compared to normotensive controls (9, 15) as well as a flattened AldAR in EHT (9). With regard to CHD compared to healthy controls, studies assessed total aldosterone from blood samples at single measurement timepoints, either assessed at an unstandardized timepoint in the morning (16), or without any further information regarding assessment timepoints (17–19). Results however remain inconclusive with elevated (16), lower (17, 18), or comparable aldosterone concentrations in CHD as compared to controls (19). Notably, to date, no study has assessed circadian aldosterone in CHD compared to healthy controls or patients with EHT using more than a single measurement timepoint, neither for salivary biologically active aldosterone, nor for total aldosterone in plasma or serum. Given that both, HT and CHD have been linked to dysregulations in the circadian rhythm of the hypothalamus-pituitary-adrenal (HPA) axis e. g (20, 21)., and as the HPA axis (co)regulates aldosterone secretion (3), an accordingly altered circadian aldosterone secretion in CHD appears plausible.

All of the following prospective studies that we identified assessed aldosterone based on a single measurement, typically obtained from a fasting morning blood sample. Prospective studies in initially healthy participants found that higher total aldosterone levels were associated with an increased risk of rising BP and the development of HT over four (22) and five years (23). Moreover, CHD incidence rates (24) and coronary calcification (25) were higher six to seven years later. Similarly, in CHD patients, higher plasma total aldosterone levels independently predicted acute ischemic events and future mortality up to 7.7 years (18, 19, 26). In myocardial infarction (MI) patients, higher plasma total aldosterone levels (mostly assessed at 24 h after MI) were associated with adverse outcomes including mortality during 0.5–5 years follow-up (27–30). Regarding the process of atherosclerosis, prospective studies found that in cardiovascular or CHD patients, plasma total aldosterone was associated with an increased atherosclerotic burden during a median follow-up of 4.7 years (19) and greater progression of carotid plaque after two years (31). Taken together, although studies are lacking that assess circadian aldosterone secretion based on repeated assessment over the day, the existing prospective evidence to date consistently points to associations between higher total (morning) aldosterone and adverse HT and CHD outcomes.

With respect to biological mechanisms underlying a potential association between higher aldosterone and adverse HT and CHD outcomes, important independent biological CHD risk factors that underly the process of atherosclerosis and thrombosis include markers of inflammation and hyperlipidemia, such as high levels of C-reactive protein (CRP) or low-density lipoprotein (LDL) (e.g (32). To the best of our knowledge, associations between aldosterone and blood lipids or inflammatory markers in humans have rarely been investigated. In two cross-sectional studies, higher plasma total aldosterone was associated with adverse lipid profiles, i.e., with higher LDL cholesterol (33) and lower high-density lipoprotein (HDL) cholesterol (33, 34). Moreover, evidence from patients with primary aldosteronism (PA) – a condition characterized by excessive aldosterone secretion – points to lower levels of total cholesterol (tChol) and triglycerides in PA (35).

Regarding inflammation, in normotensives, plasma total aldosterone was not associated with CRP (36) and an infusion study showed that intravenous aldosterone significantly increased interleukin (IL)-6 but did not affect CRP (37). Further evidence for associations between aldosterone and IL-6, CRP, and tumor necrosis factor-α (TNF-α) is provided by research in PA (36, 38, 39). To date, prospective evidence addressing associations between circadian aldosterone and independent biological CHD risk factors is lacking so far. A better understanding of the biological mechanisms underlying disease progression may have implications for long-term therapy in vulnerable populations such as patients with EHT and cardiovascular disease (CVD).

Based on the above described evidence, the first objective of our study was to cross-sectionally compare free AldAR between CHD-patients, unmedicated EHT, and healthy normotensive controls (NT). We particularly expected higher AldAR in EHT as compared to NT. Second, to obtain new mechanistic insights with respect to a potential clinical relevance of basal AldAR, we prospectively investigated whether AldAR would predict changes in major biological CHD risk factors comprising inflammatory measures and blood lipid profiles over a mean follow-up of three years. Here, we expected higher AldAR to independently predict higher increases in these biomarkers of CVD risk over time.

## 2 Methods

### 2.1 Study participants

The current investigation is part of a series of studies assessing psychoneurobiological mechanisms in EHT and CHD (21, 40, 41). Recruitment took place from 2011–2015. The study program was approved by the ethics committee of the State of Bern, Switzerland and the study protocol is in accordance with the Declaration of Helsinki. All participants provided written informed consent.

As previously described in more detail, (e.g (21), we recruited apparently healthy, medication-free HT and NT men and male patients with a diagnosis of CHD. All participants were White. Only male participants were included due to funding constraints, to minimize variability from hormonal fluctuations in women known to affect aldosterone regulation and cardiovascular risk (e.g., menstrual cycle, contraceptive use) (42–45), and to maximize methodological rigor. The present sample comprises participants who provided biologically active salivary aldosterone awakening profiles at baseline and participated in the three-year follow-up assessment.

#### 2.1.1 Recruitment and general inclusion criteria

##### 2.1.1.1 CHD-patients

We invited male patients attending the Cardiac Prevention and Rehabilitation Clinic of the Bern University Hospital, who had received their diagnosis of CHD at least six months earlier. A total of 83 patients provided awakening profile saliva samples at baseline with 60 of them having salivary aldosterone awakening profiles at baseline. 42 of the 60 CVD patients participated in the three-year follow-up assessment.

##### 2.1.1.2 Essential hypertension and normotension

We recruited apparently healthy, HT and NT men by aid of the Swiss-Red-Cross of Bern. Members of the study team accompanied the mobile blood-donation unit that routinely records BP before blood donation. Interested blood donors were given written study information asking for the following inclusion criteria: age between 18–80 years; BP either in the HT or in the NT range (see below); smoking less than five cigarettes per day; and no alcohol or illicit drug abuse. The participants were required to meet the inclusion and exclusion criteria as verified by telephone interview using an extensive health questionnaire (40, 41). In addition, to screen for potential secondary HT, we measured serum creatinine, calcium, sodium, and potassium levels in eligible HT participants, and normal ranges were verified by a board-certified internist.

#### 2.1.2 Classification of essential hypertension and normotension

EHT and NT classification was based on a two-step assessment procedure, while CHD patients were assigned *a priori* to the study groups.

##### 2.1.2.1 Home blood pressure measurement

Interested blood donors were instructed to measure BP on three days at home in a seated position using an upper arm digital blood pressure monitor (Omron M6; Omron-Healthcare-Europe B.V., Hoofdorp, Netherlands). Home BP was to be measured twice daily (once in the morning and in the evening) after a 15-min rest. HT was defined as average systolic BP ≥ 135 mmHg and/or average diastolic BP ≥ 85 mmHg and NT was defined as average home systolic BP < 135mmHg and diastolic BP < 85mmHg according to recommendations for home BP measurements (46). From the available measurements the average home BP was computed for each participant.

##### 2.1.2.2 Study blood pressure measurement

The preliminary classification as HT or NT was verified by trained personnel at the laboratory appointment with three additional BP measurements in a seated position after a 15-minute rest by means of sphygmomanometry (Omron M6; Omron-Healthcare-Europe B.V., Hoofdorp, Netherlands). We applied the regular definition of HT and classified participants as HT if their average study systolic BP was ≥ 140 mmHg and/or their average study diastolic BP was ≥ 90 mmHg (46). Participants were classified as NT if their average study systolic and diastolic BP was < 140 mmHg and < 90 mmHg, respectively. The final group assignment of medication-free participants was based on congruent home and study BP classification.

### 2.2 Design and procedure

In anticipation of the experimental session, all participants consumed a semi-standardized breakfast following written instructions and abstained from caffeine and alcohol consumption 24 h prior to their arrival at the laboratory at 8:00 h. Questionnaires were administered, and participants’ height and weight were measured. Participants received material and written instructions for saliva collection at home, before resting study BP was assessed.

To assess longitudinal changes in CHD risk factors, blood samples were collected at 11:30 h, i.e., after a fasting for 3.5 h since arrival. All participants were invited for identical blood sampling procedures taking place about 3 years later (2.95 ± 0.07).

### 2.3 Aldosterone sampling protocol

Participants were instructed to collect saliva samples on two consecutive workdays using salivette collection devices (Sarstedt, Rommelsdorf, Germany). To assess the biologically active salivary aldosterone awakening response (AldAR), five saliva samples were collected immediately after awakening (latest by 8:00 h) and 15, 30, 45, and 60 min thereafter following instructions described previously (e.g (21)). Participants were free to wake up according to their usual schedule, but no later than 8:00 h. They were instructed to remain lying in bed for the first 15 min, to refrain from eating breakfast during the first 30 min, and to avoid brushing their teeth during the first hour after awakening. Furthermore, participants were asked to refrain from eating and drinking coffee or juicy beverages 60 min prior to saliva collection. Bed-, wake-up, and accurate sampling times were assessed by self-report and complemented by electronic monitoring devices (MEMS Track Cap, Aardex, Switzerland).

### 2.4 Biochemical analyses

#### 2.4.1 Aldosterone

Participants stored their saliva samples in the refrigerator until sampling completion and then sent the collected samples to our laboratory, where saliva samples were stored until study completion at –20 °C. We analyzed AldAR in 2021 and 2022 from frozen saliva samples that were collected at baseline from 11/2011 to 02/2016. For aldosterone analyses, samples were thawed and centrifuged at 2500 g for 10 min (Heraeus Megafuge 40 R, Thermo Fisher Scientific, Langenselbold, Germany). Aldosterone concentrations were measured in duplicates using a commercially available enzyme-linked immunosorbent assay (ELISA, “Aldosterone ELISA”, RE52301, IBL International GmbH, Hamburg, Germany); lowest level of detection was 7.37 pg/ml with a linearity range from 14.14 pg/ml to 1000 pg/ml; mean inter- and intra-assay coefficients of variance were 4.7% and 5.5%, respectively.

#### 2.4.2 Prospective CHD risk assessment

We assessed prospective CHD risk by measuring changes between baseline and follow-up assessment of the following biological risk factors (1): pro-inflammatory measures interleukin (IL)-6, tumor necrosis factor alpha (TNF-α), and the acute phase protein high sensitivity C-reactive protein (hs-CRP), and (2) blood lipids profiles in terms of total cholesterol (tChol), high-density lipoprotein (HDL), and low-density lipoprotein (LDL). Blood lipids were analyzed using enzymatic colorimetric assays on a Cobas C Analyzer (Roche, Mannheim, Germany). IL-6 and TNF-α were analyzed with a high sensitivity chemiluminescence sandwich immunoassay (Meso Scale Discovery, Rockville, USA), and CRP with a high-sensitive enzyme immunoassay (ELISA, IBL Hamburg, Germany).

### 2.5 Statistical analyses

Statistical analyses were performed using SPSS (Version 30.0) statistical software packages for MacIntosh (IBM SPSS Statistics, Chicago IL, USA). All tests were two-tailed with level of significance set at *p* < .05.

A-priori power calculation (G*Power 3.1) revealed that the total sample size to detect a small effect size of *f* = .10 in a 3 (groups) by 5 (measurement points) repeated measurement ANOVA, α = .05, and an average correlation among repeated measures of *r* = .70 in cross-sectional analyses, is *N* = 93 with a power of .80, and *N* = 117 with a power of .90.

For all participants, we calculated mean arterial BP (MAP) based on the three BP study measurements by the formula MAP = (2/3 * mean study DBP) + (1/3 * mean study SBP) and body-mass-index (BMI) by the formula BMI = kg/m^2^.

For data and measures relating to aldosterone, we calculated mean values of the two sampling days. A total of 115 participants provided accurate aldosterone samples for both sampling days. In 29 participants, aldosterone data of only one day was available and we used the sampling data of that day.

Aggregated AldAR’s across the two sampling days were computed as area under the curve with respect to ground (AUC_AldAR_). tChol, HDL, LDL, IL-6, CRP, and TNF-α changes from baseline to follow-up assessment were computed as differences between follow-up and baseline assessments.

All data were tested for normal distribution and homogeneity of variance using Kolmogorov-Smirnov and Levene’s tests prior to statistical analyses. Measures showing a skewed distribution were log-transformed. While log-transformed data were used for modeling and testing, we depict untransformed data in Tables and Figures for reasons of clarity.

To compute group differences in subject characteristics we used univariate ANOVAs. Cross-sectionally, to analyze whether groups differed in AldAR secretion, we calculated repeated measures ANOVAs and ANCOVAs with repeated assessment of aldosterone, as the dependent variable and group as the independent variable. *Post-hoc* testing comprised separate testing for differences between the three groups in aldosterone levels at the separate measurement timepoints. We controlled for possible confounding effects of awakening time, and sleep duration the night before saliva sampling, in addition to age and BMI (8). *Post-hoc* testing comprised repetition of the repeated measures AN(C)OVAs with comparisons of two subject groups (i.e., HT vs. NT, CHD vs. NT, and CHD vs. HT). We applied Huynh–Feldt correction for repeated measures.

Further, we prospectively tested whether aldosterone parameters would predict future changes in CHD risk factors. We calculated multivariate analyses of covariance (MANCOVA) with prospective changes in blood lipids (LDL, HDL, tChol) and inflammatory parameters (IL-6, TNF-α, CRP) as dependent variables. As the main independent variable of interest, we entered aggregated AldAR levels (AUC_AldAR_). Covariates were entered blockwise as follows: the default set of covariates comprises age at baseline, time between baseline and follow-up assessments, and medication intake in normotensive and hypertensive individuals at follow-up (model 1). Sleep duration and wake-up time (model 2), study group (model 3), prospective changes in BMI and MAP (model 4), and BMI at baseline (model 5) were added successively as covariates to each previous model in complementary analyses. To detect the direction of MANCOVA effects, we report standardized beta coefficients by means of corresponding linear regression analyses.

## 3 Results

### 3.1 Participants’ characteristics

Demographic and medical characteristics of the 60 CHD, 40 HT, and 44 NT participants are depicted in **Table 1**. CHD-patients showed the highest age (*p* < .001), EHT and CHD-patients had a higher BMI than NT (*p* < .001), and CHD showed a longer sleep duration and later wake-up times than NT (*p* ≤ .038). EHT had the highest study systolic BP, diastolic BP, MAP, and CRP compared with NT and CHD-patients (*p* < .001). CHD patients showed lowest tChol and LDL levels (*p* < .001). On average, EHT had serum levels of creatinine, calcium, sodium, and potassium in the normal reference range, thus supporting a diagnosis of EHT. Of the 144 participants who took part in the first appointment, 47 participants dropped out resulting in a follow-up sample of *N* = 97 (see **Table 2** for follow-up characteristics, **Table 3** for baseline characteristics, and **Supplementary Table 2** for a comparison between participants who completed the follow up vs. those who dropped out). At follow-up, 12 of the 26 EHT participants reported medication use: nine received blood pressure-lowering, lipid-lowering, or anticoagulant drugs, and five were treated with other medications, such as antidiabetics or drugs for prostate symptoms (**Supplementary Table 3**).

**Table 1:** Group characteristics, aldosterone levels, and intermediate biological CHD risk factors at baseline.

Characteristics	CHD, *n* = 60	EHT, *n* = 40	NT, *n* = 44	*p*			
				NT vs. EHT vs. CHD	NT vs. EHT	NT vs. CHD	EHT vs. CHD
Age (years)	65.93 ± 1.16 (44–85)	51.80 ± 1.74 (31–74)	51.57 ± 1.81 (25–78)	**<.001**	.82	**<.001**	**<.001**
BMI (kg/m^2^)	27.83 ± 0.51 (22.60–46.44)	28.91 ± 0.63 (20.35–38.86)	25.37 ± 0.38 (19.78–30.85)	**<.001**	**<.001**	**<.001**	.18
Wake-up time (h)	6:19 ± 0:05 (4:12–8:00)	6:06 ± 0:06 (4:20–7:27), *n* = 39	5:58 ± 0:06 (4:05–7:07), *n* = 41	**.073**	.43	**.026**	.17
Sleep duration (h)	7.41 ± 0.16 (5.84–9.50), *n* = 58	7.31 ± 0.11 (6.25–9.00), *n* = 39	7.04 ± 0.13 (4.94–8.38), *n* = 41	**.083**	.11	**.038**	.58
Study BP (mmHg)							
Study SBP	142.34 ± 2.11 (95.33–187.33)	154.74 ± 1.97 (129.33–189.67)	127.25 ± 1.28 (109.33–139.67)	**<.001**	**<.001**	**<.001**	**<.001**
Study DBP	81.78 ± 1.37 (52.67–105.00)	96.72 ± 1.34 (80.00–115.00)	78.11 ± 1.09 (58.33–89.00)	**<.001**	**<.001**	.051	**<.001**
Study MAP	101.96 ± 1.46 (66.89–132.00)	116.06 ± 1.46 (103.11–139.89)	94.49 ± 1.06 (75.33–104.56)	**<.001**	**<.001**	**<.001**	**<.001**
Home BP (mmHg)							
Home SBP		145.61 ± 1.33 (132–162.33), *n* = 38	122.77 ± 0.87 (110.00–134.40)		**<.001**		
Home DBP		87.52 ± 1.12 (76.50–103.00), *n* = 38	72.39 ± 0.74 (60.83–80.00)		**<.001**		
Creatinine (μmol/L)		81.43 ± 1.60 (64–103)					
Sodium (mmol/L)		140.71 ± 0.31 (137–145), *n* = 35					
Calcium (mmol/L)		2.38 ± 0.02 (2.11–2.58), *n* = 35					
Potassium (mmol/L)		4.09 ± 0.03 (3.70–4.70), *n* = 35					
Inflammation							
IL-6 (pg/mL)	0.71 ± 0.14 (0.03–8.30), *n* = 59	0.57 ± 0.04 (0.16–1.27), *n* = 39	0.45 ± 0.03 (0.22–1.37), *n* = 43	.18	**.030**	.11	.41
TNF-α (pg/mL)	2.01 ± 0.09 (0.71–4.19), *n* = 59	1.84 ± 0.07 (1.15–3.28), *n* = 39	2.03 ± 0.11 (0.70–4.91), *n* = 43	.33	.16	.88	.18
CRP (μg/mL)	1.80 ± 0.21 (0.07–7.59), *n* = 55	3.06 ± 0.32 (0.64–8.65), *n* = 38	1.95 ± 0.30 (0.11–9.59), *n* = 36	**.003**	**.015**	.67	**<.001**
Blood lipids							
tChol (nmol/l)	4.10 ± 0.09 (2.71–5.42), *n* = 58	5.60 ± 0.14 (4.11–7.57)	5.45 ± 0.17 (3.70–7.89), *n* = 42	**<.001**	.49	**<.001**	**<.001**
LDL (nmol/l)	2.36 ± 0.07 (1.13–3.88), *n* = 58	3.79 ± 0.12 (2.33–5.51)	3.67 ± 0.15 (2.25–6.53), *n* = 42	**<.001**	.54	**<.001**	**<.001**
HDL (nmol/l)	1.40 ± 0.04 (0.92–2.40), *n* = 58	1.41 ± 0.05 (0.87–2.22)	1.52 ± 0.05 (0.91–2.21), *n* = 42	.11	.11	**.043**	.86
Aldosterone							
Awakening (pg/ml)	41.75 ± 2.01 (9.19–82.08)	58.67 ± 3.30 (31.14–143.93)	49.39 ± 2.42 (15.82–87.10)	**<.001**	**.020**	**.023**	**<.001**
15 min (pg/ml)	43.00 ± 2.10 (6.82–115.55)	63.71 ± 5.63 (21.68–258.11)	50.27 ± 2.55 (21.57–106.04)	**<.001**	**.011**	**.023**	**<.001**
30 min (pg/ml)	44.25 ± 2.49 (6.04–127.35)	59.82 ± 2.92 (31.16–109.48)	55.41 ± 2.70 (19.83–120.75)	**<.001**	.24	**.002**	**<.001**
45 min (pg/ml)	48.02 ± 3.31 (6.04–164.48)	60.36 ± 3.39 (19.30–115.19)	61.43 ± 3.09 (22.04–133.02)	**<.001**	.63	**<.001**	**.005**
60 min (pg/ml)	48.83 ± 3.35 (4.97–188.77)	60.08 ± 2.80 (21.68–103.28)	57.88 ± 2.88 (23.97–124.02)	**<.001**	.56	**.008**	**.003**

Values are *M* ± *SEM*; CHD, coronary heart disease patients; EHT, essential hypertensive individuals; NT, normotensive individuals; BMI, body mass index; DBP, diastolic blood pressure; SBP, systolic blood pressure; MAP, mean arterial blood pressure; statistical testing is based on the full sample; the first *p* corresponds to the overall comparison of the three groups. Significant values (p < .05) are highlighted in bold.

**Table 2:** Group characteristics and intermediate biological CHD risk factors at follow-up.

Characteristics	Total, *N* = 97	CHD, *n* = 42	EHT, *n* = 26	NT, *n* = 29
Time baseline to follow-up (months)	35.46 ± 0.85 (17–63)	33.52 ± 1.18 (17–58)	38.35 ± 1.64 (28–61)	35.69 ± 1.66 (21–63)
Age	60.32 ± 1.23 (31–81)	67.81 ± 1.31 (47–80)	55.00 ± 2.21 (35–77)	54.24 ± 2.19 (31–81)
BMI	27.28 ± 0.38 (20.32–40.44), *n* = 89	27.29 ± 0.49 (22.95–34.45), *n* = 34	29.49 ± 0.81 (23.77–40.44)	25.31 ± 0.48 (20.32–32.10)
SBP	138.61 ± 1.84 (102.33–181.67), *n* = 89	135.83 ± 2.62 (109.67–169.00), *n* = 34	152.33 ± 3.20 (124.33–181.67)	129.57 ± 2.41 (102.33–163.33)
DBP	82.28 ± 1.22 (55.67–112.00), *n* = 89	76.72 ± 1.51 (57.67–97.33), *n* = 34	94.08 ± 1.81 (74.67–112.00)	78.25 ± 1.47 (55.67–92.00)
Inflammation				
IL-6 (pg/mL)	0.73 ± 0.11 (0.02–10.18), *n* = 92	0.91 ± 0.25 (0.02–10.18), *n* = 40	0.72 ± 0.08 (0.27–2.25), *n* = 25	0.49 ± 0.05 (0.22–1.51), *n* = 27
TNF-α (pg/mL)	2.27 ± 0.09 (0.96–6.12), *n* = 90	2.14 ± 0.13 (0.96–4.42), *n* = 40	2.50 ± 0.23(1.31–6.12), *n* = 25	2.25 ± 0.09 (1.52–3.32), *n* = 25
CRP (μg/mL)	2.90 ± 0.37 (0.01–19.23), *n* = 85	2.76 ± 0.69 (0.01–19.23), *n* = 36	3.46 ± 0.50 (0.91–11.90), *n* = 24	2.57 ± 0.61 (0.12–14.19), *n* = 25
Blood lipids				
tChol (nmol/l)	4.87 ± 0.12 (2.81–7.81), *n* = 87	3.91 ± 0.18 (2.81–5.70), *n* = 37	5.52 ± 0.17 (3.88–6.98), *n* = 24	5.64 ± 0.17 (3.86–7.81), *n* = 26
LDL (nmol/l)	2.97 ± 0.11 (1.27–5.72), *n* = 87	2.19 ± 0.11 (1.27–3.77), *n* = 37	3.66 ± 0.16 (2.00–5.06), *n* = 24	3.43 ± 0.17 (1.33–5.72), *n* = 26
HDL (nmol/l)	1.43 ± 0.42 (0.77–3.31), *n* = 87	1.37 ± 0.06 (0.84–2.83), *n* = 37	1.36 ± 0.06 (1.01–2.20), *n* = 24	1.58 ± 0.10 (0.77–3.31), *n* = 26

Values are *M* ± *SEM*; CHD, coronary heart disease patients; EHT, essential hypertensive individuals; NT, normotensive individuals; BMI, body mass index; DBP , diastolic blood pressure; SBP, systolic blood pressure.

**Table 3:** Group characteristics, aldosterone levels, and intermediate biological CHD risk factors at baseline in follow-up participants (*n* = 97).

Characteristics	Total, *n* = 97	CHD, *n* = 42	EHT, *n* = 26	NT, *n* = 29
Age (years)	57.411.25 (27–78)	65.05 ± 1.33 (44–77)	51.92 ± 2.22 (31–74)	51.28 ± 2.24 (27–78)
BMI (kg/m^2^)	27.03 ± 0.36 (19.78–38.86)	27.23 ± 0.48 (22.60–33.80)	29.04 ± 0.77 (20.35–38.86)	24.95 ± 0.46 (19.78–29.35)
Wake-up time (h)	6:07 ± 0:04 (4:05–8:00), *n* = 93	6:18 ± 0:06 (4:12–8:00)	6:03 ± 0:08 (4:20–7:27), *n* = 25	5:54 ± 0:09 (4:05–7:07), *n* = 26
Sleep duration (h)	7.27 ± 0.09 (4.94–9.50), *n* = 92	7.39 ± 0.13 (5.84–9.50), *n* = 41	7.32 ± 0.15 (6.25–9.00), *n* = 25	7.04 ± 0.18 (4.94–8.38), *n* = 26
Study BP (mmHg)				
Study SBP	140.16 ± 1.79 (95.33–189.67)	139.04 ± 2.11 (95.99–179.00)	156.78 ± 2.65 (135.67–189.67)	126.89 ± 1.70 (109.33–139.67)
DBP	84.09 ± 1.24 (52.67–115.00)	80.23 ± 1.62 (52.67–103.50)	97.06 ± 1.80 (80.00–115.00)	78.04 ± 1.43 (58.33–89.00)
Study MAP	102.78 ± 1.35 (66.89–139.89)	96.83 ± 1.73 (66.89–121.89)	116.96 ± 1.99 (103.11–139.89)	94.32 ± 1.42 (75.33–104.56)
Home BP (mmHg)				
Home SBP			145.63 ± 1.70 (134.83–162.33), *n* = 25	123.54 ± 1.02 (110.00–134.40)
Home DBP			87.89 ± 1.46 (77.67–103.00), *n* = 25	73.37 ± 0.74 (65.67–79.33)
Creatinine (μmol/L)			81.27 ± 1.75 (66.00–99.00)	
Sodium (mmol/L)			140.55 ± 0.36 (138.00–144.00) *n* = 22	
Calcium (mmol/L)			2.38 ± 0.02 (2.11–2.58), *n* = 22	
Potassium (mmol/L)			4.06 ± 0.03 (3.70–4.30), *n* = 22	
Inflammation				
IL-6 (pg/mL)	0.61 ± 0.09 (0.03–8.30), *n* = 96	0.74 ± 0.19 (0.03–8.30)	0.58 ± 0.05 (0.27–1.25), *n* = 25	0.44 ± 0.05 (0.23–1.37)
TNF-α (pg/mL)	2.04 ± 0.07 (0.80–4.91), *n* = 96	1.94 ± 0.11 (0.80–4.19)	1.99 ± 0.09 (1.34–3.28), *n* = 25	2.24 ± 0.13 (1.43–4.91)
CRP (μg/mL)	2.03 ± 0.21 (0.07–9.59), *n* = 88	1.38 ± 0.23 (0.07–6.96), *n* = 39	3.32 ± 0.46 (0.64–8.65), *n* = 24	1.81 ± 0.41 (0.11–9.48), *n* = 25
Blood lipids				
tChol (nmol/l)	4.88 ± 0.11 (2.71–7.89), *n* = 96	4.09 ± 0.09 (2.71–5.42)	5.62 ± 0.18 (4.11–7.57)	5.39 ± 0.19 (3.70–7.89), *n* = 28
LDL (nmol/l)	3.12 ± 0.10 (1.13–5.87), *n* = 96	2.37 ± 0.09 (1.13–3.88)	3.79 ± 0.14 (2.33–5.01)	3.62 ± 0.16 (2.25–5.84), *n* = 28
HDL (nmol/l)	1.44 ± 0.03 (0.91–2.21), *n* = 96	1.40 ± 0.04 (0.92–1.97)	1.38 ± 0.05 (0.93–2.09)	1.56 ± 0.06 (0.91–2.21), *n* = 28
Aldosterone				
Awakening (pg/ml)	47.81 ± 1.60 (9.19–89.64)	41.76 ± 2.18 (9.19–78.47)	53.79 ± 2.77 (31.14–89.64)	51.22 ± 3.14 (15.82–87.10)
15 min (pg/ml)	49.64 ± 1.87 (6.82–115.55)	42.83 ± 2.60 (6.82–115.55)	60.53 ± 3.24 (35.31–99.70)	49.74 ± 3.33 (71.92–106.04)
30 min (pg/ml)	51.87 ± 2.11 (6.04–127.35)	44.39 ± 3.12 (6.04–127.35)	59.68 ± 3.99 (31.16–109.48)	55.70 ± 3.53 (19.83–120.75)
45 min (pg/ml)	55.19 ± 2.54 (6.04–164.48)	48.29 ± 4.37 (6.04–164.48)	57.21 ± 4.22 (19.30–115.19)	63.38 ± 3.77 (31.81–133.02)
60 min (pg/ml)	54.39 ± 2.34 (4.97–188.77)	50.21 ± 4.34 (4.97–188.77)	57.47 ± 3.02 (21.68–87.02)	57.67 ± 3.75 (23.97–124.02)

Values are *M* ± *SEM*; CHD, coronary heart disease patients; EHT, essential hypertensive individuals; NT, normotensive individuals; BMI, body mass index; DBP, diastolic blood pressure; SBP, systolic blood pressure; MAP, mean arterial blood pressure.

### 3.2 Aldosterone awakening response at baseline

**Figure 1:**
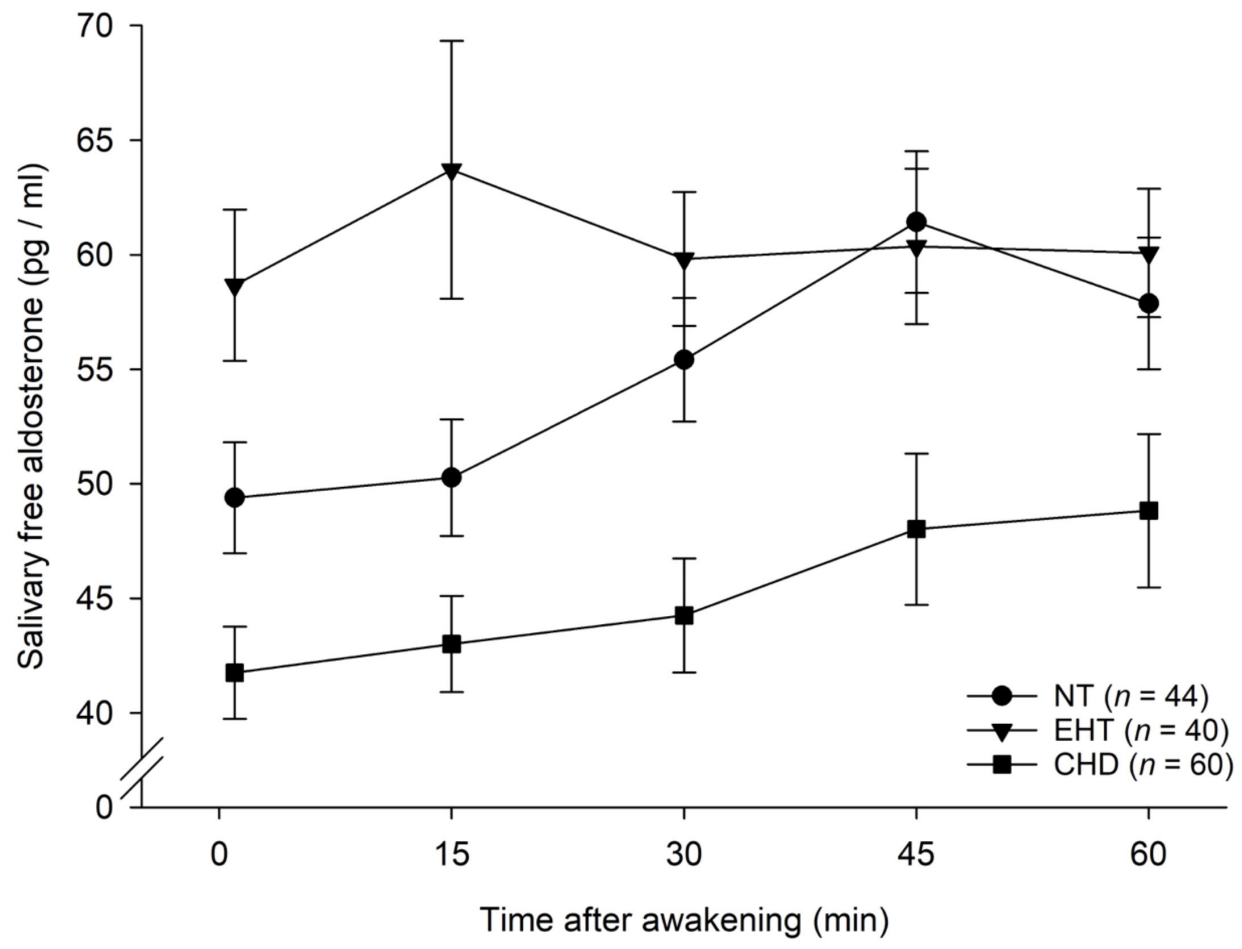
Salivary free aldosterone awakening response in essential hypertensive individuals (EHT), coronary heart disease patients (CHD) and normotensive participants (NT) (*mean* ± *SEM*).

Repeated measures AN(C)OVAs with AldAR concentrations as repeated dependent variable revealed that the three groups significantly differed in their AldAR (interaction group-by-time: *F*(7.14, 503.02) = 2.09, *p* = .042, η_*p*_^2^ = .03, *f* = 0.17; with covariates: interaction group-by-time: *p* = .37; main effect group: *F* (2, 137*)* = 6.15, *p* = .003, η_*p*_^2^ = .08, *f* = 0.30) (see **Figure 1**). Further, pairwise comparisons were performed. Comparing HT vs. CHD and NT vs. CHD revealed significantly lower AldAR in CHD as compared with the other two groups (main effect of group in HT vs. CHD: *F* (1, 98*)* = 19.20, *p* < .001, η_*p*_^2^ = .16, *f* = 0.44; with covariates: *F* (1, 94*)* = 11.18, *p* = .001, η_*p*_^2^ = .11, *f* = 0.34; main effect of group in NT vs. CHD: *F*(1, 102) = 10.58, *p* = .002, η_*p*_^2^ = .09, *f* = 0.32; with covariates: *F*(1, 98) = 3.24, *p* = .075, η_*p*_^2^ = .03, *f* = 0.18. Comparing HT vs. NT revealed higher AldAR in HT (interaction group-by-time: *F*(3.46, 283.42) = 3.59, *p* = .010, η*_p_*^2^ = .04, *f* = 0.21, see **Figure 1**; with confounders: *F*(3.60, 280.97) = 2.31, *p* = .065, η*_p_*^2^ = .03, *f* = 0.17). *Post-hoc* testing comprised repetition of AN(C)OVA analyses in each group separately. Whereas NT showed the regular AldAR (main effect of time in NT: *F*(3.54, 152.13) = 7.71, *p* < .001, η_*p*_^2^ = .15, *f* = 0.42), HT did not (main effect of time in HT: *p* = .89) but had the highest AldAR levels (see **Figure 1**). CHD showed the regular AldAR (main effect of time in CHD: *F*(3.72, 219.57) = 2.96, *p* = .024, η_*p*_^2^ = .05, *f* = 0.22) but on a lower level as compared to NT.

### 3.3 Prediction of future CHD risk by the aldosterone awakening response

MANCOVA analysis revealed that higher biologically active aldosterone levels in terms of AUC_AldAR_ overall significantly related to greater increases in *inflammatory parameters* (MANCOVA multivariate effects: model 1: *F*(3, 73) = 3.35, *p* = .024, η_*p*_^2^ = .12, *f* = 0.37, Wilk’s Λ = .88). Additional controlling for further covariates (models 2 to 5) did not alter the significance of this multivariate effect (*p*’s ≤ .032). In more detail, there was a trend for a significant association between higher AUC_AldAR_ and higher IL-6 increases (MANCOVA between-subject effects: model 1: *F*(1, 75) = 3.43, *p* = .068, η_*p*_^2^ = .04, *f* = 0.21, ß = .21, **Supplementary Figure 1**). Accordingly, in models 2 to 5 there was a trend for AUC_AldAR_ being associated with prospective changes in IL-6 (*p*’s ≤ .079). AUC_AldAR_ was not associated either with TNF-α or CRP (*p*’s ≥ .11).

Moreover, MANCOVA analysis revealed that there was a trend for an association of higher AUC_AldAR_ with higher overall increases in *blood lipids* (MANCOVA multivariate effects: model 1: *F*(3, 80) = 2.22, *p* = .093, η_*p*_^2^ = .08, *f* = 0.29, Wilk’s Λ = .92). Additional controlling for further covariates in models 2, 4, and 5 did not alter the significance of this multivariate effect (*p*’s ≤ .089). In more detail, higher AUC_AldAR_ levels were significantly associated with greater LDL and tChol increases (MANCOVA between-subject effects: model 1 LDL: *F*(1, 82) = 4.49, *p* = .037, η_*p*_^2^ = .05, *f* = 0.23, ß = .25; model 1 tChol: *F*(1, 82) = 4.75, *p* = .032, η_*p*_^2^ = .06, *f* = 0.24, ß = .26, **Supplementary Figure 1**). In models 2 to 5 there was a trend for AUC_AldAR_ levels to be associated with prospective increases in both LDL (*p*’s ≤ .067) and tChol (*p*’s ≤ .064). AUC_AldAR_ was not associated with HDL (*p*’s ≥ .31).

## 4 Discussion

To date, the role of circadian changes in aldosterone in the pathophysiology of EHT and CHD remains poorly understood. Here, we *cross-sectionally* investigated for the first time biologically active salivary AldAR levels in CHD as compared to EHT and NT controls. We found that the three groups (EHT, NT, and CHD) differed in their AldAR secretion. Whereas NT showed the regular AldAR, EHT had higher overall biologically active aldosterone levels but a flattened AldAR, both independent of confounders. This result aligns with previous findings (9). The increased morning biologically active aldosterone levels in combination with a flattened AldAR in unmedicated EHT may indicate a generally altered RAAS activity with underlying alteration in hypothalamic–pituitary–adrenal (HPA) axis and sympathetic–adrenomedullary system activity. Independent of confounders, CHD patients showed the regular AldAR but on a lower level as compared to NT. We attribute this finding to the medication intake in the CHD group, with the vast majority of patients taking ACE-inhibitors and other RAAS affecting medication (see **Supplementary Table 1**).

Our results suggest that dysregulation in terms of an increased AldAR may represent an early indicator for increased cardiovascular risk. However, the mechanisms underlying the observed alteration in AldAR in both EHT and CHD are unclear. A possible explanation may be (former) chronic stress experiences. Chronic stress was proposed to play a role in both HT and CHD development that has been associated with altered circadian HPA-axis activity (47, 48). Notably, there is also evidence for altered aldosterone levels in conditions of psychological chronic stress (e.g., social isolation or mental disorders) e. g (49–53). In line with the allostatic load concept by McEwen (54) it is conceivable that under conditions of chronic stress repeated increases in aldosterone may accumulate over time to chronically elevated levels, in particular after awakening, and consequently elevated CHD risk. Also, the sympathetic overload with manifest HT (55) may induce stress-like physiological reactions that may maintain, or add to chronic stress reactions.

Furthermore, to shed light on the clinical relevance of circadian aldosterone, we *prospectively* investigated whether AldAR would predict changes in major biological CHD risk factors comprising inflammatory measures and blood lipid profiles over a mean follow-up of three years. Notably, we controlled, among others, for medication intake and we investigated the associations for the first time in a longitudinal study. We found higher AldAR in terms of AUC_AldAR_, to predict greater increases in inflammatory markers and blood lipids. Specifically, higher AUC_AldAR_ predicted a trend to significantly higher independent IL-6 increases, but not TNF-α or CRP. For example, studies found higher levels of IL-6, CRP, and TNF-α in patients with PA than in NT or patients with EHT (36, 38, 39) and plasma IL-6 levels were found to be correlated with higher 24-hour urinary aldosterone in PA (39). Our results correspond with findings from an infusion study that found increases in IL-6 but not in CRP after aldosterone administration (37). Higher biologically active aldosterone levels can lead to increased IL-6 levels through several mechanisms. For example, aldosterone-induced activation of the mineralocorticoid receptor (MR) enhances the production of pro-inflammatory cytokines, including IL-6, by promoting nuclear factor-kappa B (NF-κB) signaling (37, 56–60). Further, aldosterone increases the production of reactive oxygen species (ROS) and ROS further activate NF-κB that enhances IL-6 gene expression (61). Moreover, aldosterone can reduce nitric oxide availability which in turn promotes IL-6 release (62). Moreover, adipose tissue may play a role (see below).

Regarding blood lipids, higher AldAR predicted increases in LDL cholesterol and tChol, however, there was no association with HDL cholesterol. Notably, controlling for confounders reduced the level of significance towards a trend. Our results correspond with findings from a cross-sectional study which found higher plasma total aldosterone to be associated with higher LDL cholesterol (33). The mechanisms underlying the association between aldosterone and lipid concentration increases are not well understood; however, elevated aldosterone levels may contribute to dyslipidemia, including higher tChol and LDL cholesterol. Studies that suggest a link between aldosterone and the development of adipose tissue could provide an explanation. For example, aldosterone was shown to promote adipose tissue growth (63–66) and the MR plays an important role in adipose cell differentiation (64, 67). Adipose tissue as a major site for cholesterol storage (68, 69) could in turn release not only pro-inflammatory cytokines including IL-6 (70) but also tChol and LDL cholesterol into the blood. Furthermore, elevated aldosterone concentrations over the long term may induce renal disease with concomitant proteinuria (71) that might stimulate an increased LDL synthesis (72). Additionally, lower levels of tChol have been reported in PA patients compared to those with EHT, potentially due to mechanisms such as glomerular hyperfiltration or the confounding effects of thiazide use (35). Notably, most of the hitherto available literature suggests that adipose tissue contributes to stimulation of aldosterone release (73). In contrast to previous (cross-sectional) findings (34, 33), aldosterone was not associated with HDL cholesterol in our sample. Notably, it should be emphasized that we measured the biologically active AldAR from saliva within 60 min after awakening, in contrast to previous studies (33, 34, 37) which assessed total aldosterone in blood using single time-point measurements. To further investigate this aspect, future research is needed that includes parallel assessment of AldAR from both plasma and saliva to capture not only the bound but also the biologically active free fraction of aldosterone.

In addition to the inflammatory and lipid parameters analyzed in our study, there are other mechanisms (e.g., endothelial dysfunction or oxidative stress) by which aldosterone may contribute to cardiovascular risk. Studies have demonstrated that aldosterone can reduce endothelial nitric oxide synthase activity and thereby lower nitric oxide bioavailability (e.g (74)). Moreover, aldosterone can promote superoxide generation via nicotinamide adenine dinucleotide phosphate (NADPH) oxidase activation (75) and can reduce antioxidant defenses, including enzymes such as superoxide dismutase (76). Although we did not assess these markers in the present work, they should be considered in future studies to provide a more comprehensive understanding of the multifaceted cardiovascular effects of aldosterone.

Clinical implications of our study may include supplementing standard diagnostic and monitoring approaches for EHT and CHD with the assessment of awakening salivary biologically active aldosterone profiles in primary care. Routine measurement of these profiles could serve as an early indicator of future disease risk, and elevated awakening aldosterone levels may warrant closer medical attention or targeted treatment. In this context, it is important to note that aldosterone concentrations are influenced by posture (e.g (77)). Clinical measurements are typically standardized after rest and after a defined period of upright posture, whereas samples taken at home may vary considerably due to preceding posture changes and movement. These physiological principles should be considered when interpreting and comparing results across different settings. Limitations of our study include the relatively high drop-out rate that we mainly attribute to logistical challenges, particularly the change in study sites between two institutions during the course of the study. Furthermore, while RAAS-modulating drugs may influence AldAR, the heterogeneity of medications in our cohort and the limited sample size did not allow to control for specific medication categories, which should be addressed in future studies. Notably, excluding the two CHD patients who were taking MRAs from the analyses of aldosterone profiles did not significantly change the results (data not shown). A major limitation of our study is that we included middle-aged men of relatively high socioeconomic status. This substantially restricts the generalizability of our findings to women, older participants, or clinical populations. Future studies are needed to examine whether our findings also apply to women and participants with differing socioeconomic status. In particular, hormonal fluctuations in women may affect aldosterone regulation (e.g (42, 43)) and future studies should include female participants to assess potential sex-specific differences in AldAR and cardiovascular risk. In our study, AldAR was assessed from saliva samples collected on two consecutive days to account for day-to-day variability. We recognize, however, that this limits feasibility in clinical practice. Future studies should therefore investigate simplified protocols, and compare their diagnostic performance to frequent repeated sampling. Moreover, we measured salivary aldosterone by means of ELISA and thus using an antibody-based immunoassay. As compared to liquid chromatography-mass spectrometry (LC-MS/MS), antibody-based assays usually have a lower specificity as potential cross-reactions cannot be completely excluded, but allow for a higher throughput. Our findings thus warrant replication in future studies using analytic methods other than immunoassays. A further limitation of our study is the lack of information on participants’ salt intake, which could not be controlled under the real-life conditions of home-based data collection. In addition, assessment of plasma renin levels in parallel to biologically active salivary aldosterone was not feasible in the salivary assessment in participants’ homes. Further, licorice intake was not explicitly assessed within the study protocol. We therefore cannot exclude the possibility that some participants may have consumed licorice without reporting it. This should be acknowledged as a potential limitation, as unrecognized licorice intake could have influenced blood pressure or aldosterone concentrations (e.g (78, 79)). Strengths of our study comprise the use of MEMS caps combined with self-recording of sampling times allowing us to ensure the adherence to the study protocol. We applied a highly standardized procedure with frequent sampling times in the first hour after awakening in participants’ natural environment. Further, when assessing biologically active salivary aldosterone, we controlled for important potentially confounding variables including waking time and sleep duration and saliva samples were assessed on two consecutive days (80).

Taken together, we found evidence for altered biologically active AldAR in CHD and EHT as compared to NT. Our results moreover suggest that higher biologically active AldAR seems to predict poorer cardiovascular health, in particular of atherogenic lipids and basal inflammation in terms of IL-6. Higher basal biologically active AldAR activity, either without or despite medication intake, may therefore play a role in the pathogenesis of atherosclerosis.

## Data Availability

The raw data supporting the conclusions of this article will be made available by the authors, without undue reservation.

## References

[1] Virani SS Alonso A Aparicio HJ Benjamin EJ Bittencourt MS Callaway CW . Heart disease and stroke statistics—2021 update: a report from the American Heart Association. Circulation. (2021) 143:e254–743. doi: 10.1161/CIR.0000000000000950, PMID: 33501848 PMC13036842

[2] Messerli FH Williams B Ritz E . Essential hypertension. Lancet. (2007) 370:591–603. doi: 10.1016/S0140-6736(07)61299-9, PMID: 17707755

[3] Connell JM Davies E . The new biology of aldosterone. J Endocrinol. (2005) 186:1–20. doi: 10.1677/joe.1.06017, PMID: 16002531

[4] Zipser RD Meidar V Horton R . Characteristics of aldosterone binding in human plasma. JCEM. (1980) 50:158–62. doi: 10.1210/jcem-50-1-158, PMID: 7350179

[5] Manolopoulou J Mulatero P Maser-Gluth C Rossignol P Spyroglou A Vakrilova Y . Saliva as a medium for aldosterone measurement in repeated sampling studies. Steroids. (2009) 74:853–8. doi: 10.1016/j.steroids.2009.05.006, PMID: 19481102

[6] Gideon A Sauter C Fieres J Berger T Renner B Wirtz PH . Kinetics and interrelations of the renin aldosterone response to acute psychosocial stress: a neglected stress system. JCEM. (2020) 105:e762–e73. doi: 10.1210/clinem/dgz190, PMID: 31711229 PMC7034950

[7] Few J Unwin R Carmichael D James V . Diurnal fluctuation in saliva aldosterone concentration. J Steroid Biochem. (1987) 26:265–71. doi: 10.1016/0022-4731(87)90081-1, PMID: 3560941

[8] Gideon A Sauter C Deuber J Grünewald J Wirtz PH . Aldosterone secretion during the day: salivary aldosterone awakening response and daytime levels. Psychoneuroendocrinology. (2022) 139:105685. doi: 10.1016/j.psyneuen.2022.105685, PMID: 35202970

[9] Gideon A von Känel R Degroote C Thomas L Zuccarella-Hackl C Wiest R . Increased daytime and awakening salivary free aldosterone in essential hypertensive men. Front Cardiovasc Med. (2024) 11:1335329. doi: 10.3389/fcvm.2024.1335329, PMID: 38984356 PMC11231427

[10] Upton TJ Zavala E Methlie P Kämpe O Tsagarakis S Øksnes M . High-resolution daily profiles of tissue adrenal steroids by port able automated collection. Sci Transl Med. (2023) 15:eadg8464. doi: 10.1126/scitranslmed.adg8464, PMID: 37343084

[11] Stern N Sowers JR McGinty D Beahm E Littner M Catania R . Circadian rhythm of plasma renin activity in older normal and essential hypertensive men: relation with inactive renin, aldosterone, cortisol and REM sleep. J Hypertens. (1986) 4:543–50. doi: 10.1097/00004872-198610000-00005, PMID: 3540117

[12] Portaluppi F Bagni B degli Uberti E Montanari L Cavallini R Trasforini G . Circadian rhythms of atrial natriuretic peptide, renin, aldosterone, cortisol, blood pressure and heart rate in normal and hypertensive subjects. J Hypertens. (1990) 8:85–95. doi: 10.1097/00004872-199001000-00013, PMID: 2157761

[13] Portaluppi F Trasforini G Margutti A Vergnani L Ambrosio MR Rossi R . Circadian rhythm of calcitonin gene-related peptide in uncomplicated essential hypertension. J Hypertens. (1992) 10:1227–34. doi: 10.1097/00004872-199210000-00017, PMID: 1335005

[14] Cugini P Manconi R Serdoz R Mancini A Meucci T Scavo D . Rhythm characteristics of plasma renin, aldosterone and cortisol in five subtypes of mesor-hypertension. J Endocrinol Invest. (1980) 3:143–7., PMID: 6248590 10.1007/BF03348241

[15] Manolopoulou J Gerum S Mulatero P Rossignol P Plouin P-F Reincke M . Salivary aldosterone as a diagnostic aid in primary aldosteronism. Horm Metab Res. (2010) 42:400–5. doi: 10.1055/s-0030-1248287, PMID: 20217632

[16] Karolczak K Kubalczyk P Glowacki R Pietruszynski R Watala C . Aldosterone modulates blood homocysteine and cholesterol in coronary artery disease patients–a possible impact on atherothrombosis? Physiol Res. (2018) 67:197–207., PMID: 29303611 10.33549/physiolres.933668

[17] Sablik Z Samborska-Sablik A Goch JH . Concentrations of adrenal steroids and sex hormones in postmenopausal women suffering from coronary artery disease. Polski Merkuriusz Lekarski: Organ Polskiego Towarzystwa Lekarskiego. (2008) 25:326–9., PMID: 19145930

[18] Tomaschitz A Pilz S Ritz E Meinitzer A Boehm BO März W . Plasma aldosterone levels are associated with increased cardiovascular mortality: the Ludwigshafen Risk and Cardiovascular Health (LURIC) study. Eur Heart J. (2010) 31:1237–47. doi: 10.1093/eurheartj/ehq019, PMID: 20200015

[19] Hillaert MA Lentjes EG Kemperman H van der Graaf Y Nathoe HM Beygui F . Aldosterone, atherosclerosis and vascular events in patients with stab le coronary artery disease. Int J Cardiol. (2013) 167:1929–35. doi: 10.1016/j.ijcard.2012.05.034, PMID: 22727970

[20] Wirtz PH von Känel R Emini L Ruedisueli K Groessbauer S Maercker A . Evidence for altered hypothalamus–pituitary–adrenal axis functioning in systemic hypertension: Blunted cortisol response to awakening and lower negative feedback sensitivity. Psychoneuroendocrinology. (2007) 32:430–6. doi: 10.1016/j.psyneuen.2007.02.006, PMID: 17433557

[21] Degroote C von Känel R Thomas L Zuccarella-Hackl C Messerli-Bürgy N Saner H . Lower diurnal HPA-axis activity in male hypertensive and coronary heart disease patients predicts future CHD risk. Front Endocrinol. (2023) 14:1080938. doi: 10.3389/fendo.2023.1080938, PMID: 36967749 PMC10036761

[22] Buglioni A Cannone V Sangaralingham SJ Heublein DM Scott CG Bailey KR . Aldosterone predicts cardiovascular, renal, and metabolic disease in the general community: a 4-year follow-up. J Am Heart Assoc. (2015) 4:e002505. doi: 10.1161/JAHA.115.002505, PMID: 26702078 PMC4845260

[23] Meneton P Galan P Bertrais S Heudes D Hercberg S Menard J . High plasma aldosterone and low renin predict blood pressure increase and hypertension in middle-aged Caucasian populations. J Hum Hypertens. (2008) 22:550–8. doi: 10.1038/jhh.2008.27, PMID: 18449201

[24] Joseph JJ Echouffo-Tcheugui JB Kalyani RR Yeh H-C Bertoni AG Effoe VS . Aldosterone, renin, cardiovascular events, and all-cause mortality among African Americans: the Jackson Heart Study. JACC: Heart Failure. (2017) 5:642–51., PMID: 28822744 10.1016/j.jchf.2017.05.012 PMC5705009

[25] Inoue K Goldwater D Allison M Seeman T Kestenbaum BR Watson KE . Serum aldosterone concentration, blood pressure, and coronary artery calcium: the multi-ethnic study of atherosclerosis. Hypertension. (2020) 76:113–20. doi: 10.1161/HYPERTENSIONAHA.120.15006, PMID: 32418495 PMC10681368

[26] Ivanes F Susen S Mouquet F Pigny P Cuilleret F Sautiere K . Aldosterone, mortality, and acute ischaemic events in coronary artery disease patients outside the setting of acute myocardial infarction or heart failure. Eur Heart J. (2012) 33:191–202. doi: 10.1093/eurheartj/ehr176, PMID: 21719456

[27] Palmer BR Pilbrow AP Frampton CM Yandle TG Skelton L Nicholls MG . Plasma aldosterone levels during hospitalization are predictive of survival post-myocardial infarction. Eur Heart J. (2008) 29:2489–96. doi: 10.1093/eurheartj/ehn383, PMID: 18757359

[28] Beygui F Collet J-P Benoliel J-J Vignolles N Dumaine R Bartheíleímy O . High plasma aldosterone levels on admission are associated with death in patients presenting with acute ST-elevation myocardial infarction. Circulation. (2006) 114:2604–10. 10.1161/CIRCULATIONAHA.106.634626 17116769

[29] Yuyun MF Jutla SK Quinn PA Ng LL . Aldosterone predicts major adverse cardiovascular events in patients with acute myocardial infarction. Heart Asia. (2012) 4:102–7. doi: 10.1136/heartasia-2012-010129, PMID: 27326041 PMC4832614

[30] Beygui F Montalescot G Vicaut E Rouanet S Van Belle E Baulac C . Aldosterone and long-term outcome after myocardial infarction: a substudy of the French nationwide Observatoire sur la Prise en charge hospitaliere, l’Evolution a un an et les caRacteristiques de patients presentant un infArctus du myocarde avec ou sans onde Q (OPERA) study. Am Heart J. (2009) 157:680–7. doi: 10.1016/j.ahj.2008.12.013, PMID: 19332195

[31] de Rita O Hackam DG Spence JD . Effects of aldosterone on human atherosclerosis: plasma aldosterone and progression of carotid plaque. Can J Cardiol. (2012) 28:706–11. doi: 10.1016/j.cjca.2012.04.014, PMID: 22717248

[32] Rafieian-Kopaei M Setorki M Doudi M Baradaran A Nasri H . Atherosclerosis: process, indicators, risk factors and new hopes. Int J Prev Med. (2014) 5:927., PMID: 25489440 PMC4258672

[33] Hannich M Wallaschofski H Nauck M Reincke M Adolf C Völzke H . Physiological aldosterone concentrations are associated with alterations of lipid metabolism: observations from the general population. Int J Endocrinol. (2018) 2018:4128174. doi: 10.1155/2018/4128174, PMID: 29780416 PMC5892232

[34] Goodfriend TL Egan B Stepniakowski K Ball DL . Relationships among plasma aldosterone, high-density lipoprotein cholesterol, and insulin in humans. Hypertension. (1995) 25:30–6. doi: 10.1161/01.HYP.25.1.30, PMID: 7843750

[35] Manosroi W Phudphong P Atthakomol P Phimphilai M . The differences of serum lipid profiles between primary aldosteronism and essential hypertension: a meta-analysis and systematic review. BMC Endocr Disord. (2022) 22:217. doi: 10.1186/s12902-022-01135-y, PMID: 36045354 PMC9429522

[36] Grotevendt A Wallaschofski H Reincke M Adolf C Quinkler M Nauck M . Associations of aldosterone and renin concentrations with inflammation—the Study of Health in Pomerania and the German Conn’s Registry. Endocrine. (2017) 57:298–307. doi: 10.1007/s12020-017-1348-8, PMID: 28638984

[37] Luther JM Gainer JV Murphey LJ Yu C Vaughan DE Morrow JD . Angiotensin II induces interleukin-6 in humans through a mineralocorticoid receptor–dependent mechanism. Hypertension. (2006) 48:1050–7. doi: 10.1161/01.HYP.0000248135.97380.76, PMID: 17043157

[38] Wu C Zhang H Zhang J Xie C Fan C Zhang H . Inflammation and fibrosis in perirenal adipose tissue of patients with aldosterone-producing adenoma. Endocrinology. (2018) 159:227–37. doi: 10.1210/en.2017-00651, PMID: 29059354

[39] Chou C-H Hung C-S Liao C-W Wei L-H Chen C-W Shun C-T . IL-6 trans-signalling contributes to aldosterone-induced cardiac fibrosis. Cardiovasc Res. (2018) 114:690–702. doi: 10.1093/cvr/cvy013, PMID: 29360942

[40] Zuccarella-Hackl C von Känel R Thomas L Hauser M Kuebler U Widmer HR . Macrophage superoxide anion production in essential hypertension: associations with biological and psychological cardiovascular risk factors. Psychosom Med. (2016) 78:750–7. doi: 10.1097/PSY.0000000000000324, PMID: 27187852

[41] Zuccarella-Hackl C Von Känel R Thomas L Kuebler P Schmid J-P Mattle HP . Higher macrophage superoxide anion production in coronary artery disease (CAD) patients with Type D personality. Psychoneuroendocrinology. (2016) 68:186–93. doi: 10.1016/j.psyneuen.2016.02.031, PMID: 26994482

[42] Dos Santos PA de Oliveira AM Alves CQ Souza Filho CF Ladeia AMT Petto J . Renin-angiotensin-aldosterone system in women using combined oral contraceptive: a systematic review. Rev Bras Ginecologia e Obstetrícia/RBGO Gynecology Obstetrics. (2022) 44:710–8. doi: 10.1055/s-0042-1745790, PMID: 35724684 PMC9948294

[43] Chapman AB Zamudio S Woodmansee W Merouani A Osorio F Johnson A . Systemic and renal hemodynamic changes in the luteal phase of the menstrual cycle mimic early pregnancy. Am J Physiology-Renal Physiol. (1997) 273:F777–F82. doi: 10.1152/ajprenal.1997.273.5.F777, PMID: 9374841

[44] Capri Workshop Group. Hormones ESHRE . and cardiovascular health in women. Hum Reprod Update. (2006) 12:483–97. doi: 10.1093/humupd/dml028, PMID: 16807276

[45] Dos Santos RL da Silva FB Ribeiro RF Jr. Stefanon I . Sex hormones in the cardiovascular system. Horm Mol Biol Clin Investig. (2014) 18:89–103. doi: 10.1515/hmbci-2013-0048, PMID: 25390005

[46] Williams B Mancia G Spiering W Agabiti Rosei E Azizi M Burnier M . 2018 ESC/ESH Guidelines for the management of arterial hypertension: The Task Force for the management of arterial hypertension of the European Society of Cardiology (ESC) and the European Society of Hypertension (ESH). Eur Heart J. (2018) 39:3021–104. doi: 10.1093/eurheartj/ehy339, PMID: 30165516

[47] McEwen BS . Stress, adaptation, and disease: Allostasis and allostatic load. In: Molecular aspects, integrative systems, and clinical advances. Annals of the New York Academy of Sciences. New York Academy of Sciences, New York, NY, US (1998). p. 33–44. 10.1111/j.1749-6632.1998.tb09546.x 9629234

[48] Yao B-C Meng L-B Hao M-L Zhang Y-M Gong T Guo Z-G . Chronic stress: a critical risk factor for atherosclerosis. J Int Med Res. (2019) 47:1429–40. doi: 10.1177/0300060519826820, PMID: 30799666 PMC6460614

[49] Terock J Hannemann A Janowitz D Völzke H Nauck M Freyberger H-J . Living alone and activation of the renin-angiotensin-aldosterone-system: Differential effects depending on alexithymic personality features. J Psychosom Res. (2017) 96:42–8. doi: 10.1016/j.jpsychores.2017.03.007, PMID: 28545792

[50] Häfner S Baumert J Emeny RT Lacruz ME Bidlingmaier M Reincke M . To live alone and to be depressed, an alarming combination for the renin–angiotensin–aldosterone-system (RAAS). Psychoneuroendocrinology. (2012) 37:230–7. doi: 10.1016/j.psyneuen.2011.06.007, PMID: 21742440

[51] Maillet A Gunga HC Gauquelin G Fortrat JO Hope A Røcker L . Effects of 28-day isolation (ESA-ISEMSI’90) on blood pressure and blood volume regulating hormones. Aviat Space Environ Med. (1993) 64:287–94. 8476368

[52] Kapsdorfer D Hlavacova N Vondrova D Argalasova L Sevcikova L Jezova D . Neuroendocrine response to school load in prepubertal children: focus on trait anxiety. Cell Mol Neurobiol. (2018) 38:155–62. doi: 10.1007/s10571-017-0544-7, PMID: 28861683 PMC11481945

[53] Terock J Hannemann A Janowitz D van der Auwera S Bahls M Völzke H . Differential activation of the renin-angiotensin-aldosterone-system in response to childhood and adulthood trauma. Psychoneuroendocrinology. (2019) 107:232–40. doi: 10.1016/j.psyneuen.2019.05.026, PMID: 31174161

[54] McEwen BS . Protective and damaging effects of stress mediators. N Eng J Med. (1998) 338:171–9. doi: 10.1056/NEJM199801153380307, PMID: 9428819

[55] Manolis AJ Poulimenos LE Kallistratos MS Gavras I . Sympathetic overactivity in hypertension and cardiovascular disease. Curr Vasc Pharmacol. (2014) 12:4–15. doi: 10.2174/15701611113119990140, PMID: 23905597

[56] Luther JM Fogo AB . The role of mineralocorticoid receptor activation in kidney inflammation and fibrosis. Kidney Int Suppl. (2022) 12:63–8. doi: 10.1016/j.kisu.2021.11.006, PMID: 35529087 PMC9073221

[57] Leroy V De Seigneux S Agassiz V Hasler U Rafestin-Oblin M-E Vinciguerra M . Aldosterone activates NF-κB in the collecting duct. J Am Soc Nephrol. (2009) 20:131–44. doi: 10.1681/ASN.2008020232, PMID: 18987305 PMC2615729

[58] Sanz-Rosa D Cediel E de las Heras N Miana M Balfagón G Lahera V . Participation of aldosterone in the vascular inflammatory response of spontaneously hypertensive rats: role of the NFκB/IκB system. J Hypertens. (2005) 23:1167–72. doi: 10.1097/01.hjh.0000170379.08214.5a, PMID: 15894892

[59] Fiebeler A Schmidt F Moller DN Park J-K Dechend R Bieringer M . Mineralocorticoid receptor affects AP-1 and nuclear factor-κB activation in angiotensin II–induced cardiac injury. Hypertension. (2001) 37:787–93. doi: 10.1161/01.HYP.37.2.787, PMID: 11230374

[60] Brasier AR . The nuclear factor-κB–interleukin-6 signalling pathway mediating vascular inflammation. Cardiovasc Res. (2010) 86:211–8. doi: 10.1093/cvr/cvq076, PMID: 20202975 PMC2912657

[61] Miyata K Rahman M Shokoji T Nagai Y Zhang G-X Sun G-P . Aldosterone stimulates reactive oxygen species production through activation of NADPH oxidase in rat mesangial cells. J Am Soc Nephrol. (2005) 16:2906–12. doi: 10.1681/ASN.2005040390, PMID: 16135774

[62] Fels J Oberleithner H Kusche-Vihrog K . Menage a trois: aldosterone, sodium and nitric oxide in vascular endothelium. Biochim Biophys Acta Mol Basis Dis. (2010) 1802:1193–202. doi: 10.1016/j.bbadis.2010.03.006, PMID: 20302930

[63] Devenport L Torres A Murray C . Effects of aldosterone and deoxycorticosterone on food intake and body weight. Behav Neurosci. (1983) 97:667–9. doi: 10.1037/0735-7044.97.4.667, PMID: 6615643

[64] Feraco A Armani A Mammi C Fabbri A Rosano GM Caprio M . Role of mineralocorticoid receptor and renin–angiotensin–aldosterone system in adipocyte dysfunction and obesity. J Steroid Biochem Mol Biol. (2013) 137:99–106. doi: 10.1016/j.jsbmb.2013.02.012, PMID: 23454117

[65] Rondinone CM Rodbard D Baker M . Aldosterone stimulated differentiation of mouse 3T3-L1 cells into adipocytes. Endocrinology. (1993) 132:2421–6. doi: 10.1210/en.132.6.2421, PMID: 8504747

[66] Devenport L Goodwin K Hopkins P . Continuous infusion of aldosterone: correlates of body weight gain. Pharmacol Biochem Behav. (1985) 22:707–9. doi: 10.1016/0091-3057(85)90517-9, PMID: 4011633

[67] Caprio M Feve B Claës A Viengchareun S Lombes M Zennaro M-C . Pivotal role of the mineralocorticoid receptor in corticosteroid-induced adipogenesis. FASEB J. (2007) 21:2185–94. doi: 10.1096/fj.06-7970com, PMID: 17384139

[68] Krause BR Hartman AD . Adipose tissue and cholesterol metabolism. J Lipid Res. (1984) 25:97–110. doi: 10.1016/S0022-2275(20)37830-5 6368715

[69] Chui PC Guan H-P Lehrke M Lazar MA . PPARγ regulates adipocyte cholesterol metabolism via oxidized LDL receptor 1. J Clin Invest. (2005) 115:2244–56. doi: 10.1172/JCI24130, PMID: 16007265 PMC1172230

[70] Coppack SW . Pro-inflammatory cytokines and adipose tissue. Proc Nutr Soc. (2001) 60:349–56. doi: 10.1079/PNS2001110, PMID: 11681809

[71] Epstein M . Aldosterone as a mediator of progressive renal disease: pathogenetic and clinical implications. Am J Kidney Dis. (2001) 37:677–88. doi: 10.1016/S0272-6386(01)80115-3, PMID: 11273866

[72] Trevisan R Dodesini AR Lepore G . Lipids and renal disease. J Am Soc Nephrol. (2006) 17:S145–S7. doi: 10.1681/ASN.2005121320, PMID: 16565240

[73] Schütten MT Houben AJ de Leeuw PW Stehouwer CD . The link between adipose tissue renin-angiotensin-aldosterone system signaling and obesity-associated hypertension. Physiology. (2017) 32:197–209. doi: 10.1152/physiol.00037.2016, PMID: 28404736

[74] Kirsch T Beese M Wyss K Klinge U Haller H Haubitz M . Aldosterone modulates endothelial permeability and endothelial nitric oxide synthase activity by rearrangement of the actin cytoskeleton. Hypertension. (2013) 61:501–8. doi: 10.1161/HYPERTENSIONAHA.111.196832, PMID: 23213194

[75] Iwashima F Yoshimoto T Minami I Sakurada M Hirono Y Hirata Y . Aldosterone induces superoxide generation via Rac1 activation in endothelial cells. Endocrinology. (2008) 149:1009–14. doi: 10.1210/en.2007-0864, PMID: 18079208

[76] Tsai C-H Pan C-T Chang Y-Y Peng S-Y Lee P-C Liao C-W . Aldosterone excess induced mitochondria decrease and dysfunction via mineralocorticoid receptor and oxidative stress *in vitro* and *in vivo*. Biomedicines. (2021) 9:946. doi: 10.3390/biomedicines9080946, PMID: 34440149 PMC8392669

[77] Mlynarik M Makatsori A Dicko I Hinghofer-Szalkay H Jezova D . Postural changes associated with public speech tests lead to mild and selective activation of stress hormone release. J Physiol Pharmacol. (2007) 58:95–103., PMID: 17440229

[78] Sabbadin C Bordin L Donà G Manso J Avruscio G Armanini D . Licorice: from pseudohyperaldosteronism to therapeutic uses. Front Endocrinol. (2019) 10:484. doi: 10.3389/fendo.2019.00484, PMID: 31379750 PMC6657287

[79] Deutch MR Grimm D Wehland M Infanger M Krüger M . Bioactive candy: effects of licorice on the cardiovascular system. Foods. (2019) 8:495. doi: 10.3390/foods8100495, PMID: 31615045 PMC6836258

[80] Stalder T Kirschbaum C Kudielka BM Adam EK Pruessner JC Wüst S . Assessment of the cortisol awakening response: Expert consensus guidelines. Psychoneuroendocrinology. (2016) 63:414–32. doi: 10.1016/j.psyneuen.2015.10.010, PMID: 26563991

