# Supplementary material for: Salivary free aldosterone awakening response and cardiovascular risk in men with CHD, hypertension, and healthy controls
Source: Front Endocrinol (Lausanne). 2026 Jan 5;16:1655896. doi: 10.3389/fendo.2025.1655896 (PMC12812740; doi:10.3389/fendo.2025.1655896)
Supplement: Supplementary file 1 [file DataSheet1.pdf]

Supplementary Table 1

Medication intake in CHD patients (n = 60) at baseline

Medication	Number of Patients
RAAS-modulating medication	
At least one of the following medications: ACE inhibitors, AT-II receptor antagonists, aldosterone antagonists	46
At least one of the following medications: ACE inhibitors, AT-II receptor antagonists, aldosterone antagonists, beta blockers	55
All medication	
ACE inhibitors	24
AT-II receptor antagonists	22
Aldosterone antagonists	2
Alpha-1 blockers	4
Alpha and beta blockers	1
Beta blockers	37
Antiarrhythmics	2
Anticoagulants	58
Antiplatelets	56
Lipid lowering drugs	56
Diuretics	21
Antidiabetics	3
Insulin	1
Antiepileptics	0
Antiparkinsonian drugs	2
Antidepressants	2
Others	24

Note. CHD = coronary heart disease patients.

Supplementary Table 2

Group characteristics, aldosterone levels, and intermediate biological CHD risk factors at baseline in participants who completed the follow up vs. participants who dropped out

	participants who completed the follow-up, *n* = 97	participants who dropped out, *n* = 47	*p*
Age (years)	57.41 ± 1.25 (27–78)	58.04 ± 1.94 (25–85)	.88
BMI (kg/m^2)	27.03 ± 0.36 (19.78–38.86)	28.09 ± 0.63 (21.93–46.44)	.12
Wake-up time (h)	6:07 ± 0:04 (4:05–8:00), *n* = 93	6:14 ± 0:06 (4:42–8:00)	.44
Sleep duration (h)	7.27 ± 0.09 (4.94–9.50), *n* = 92	7.27 ± 0.12 (5.98–9.50), *n* = 46	.99
Study BP (mmHg)			
Study SBP	140.16 ± 1.79 (95.33–189.67)	143.26 ± 2.21 (119.67–187.33)	.30
Study DBP	84.09 ± 1.24 (52.67–115.00)	86.29 ± 1.58 (62.67–108.00)	.30
Study MAP	102.78 ± 1.35 (66.89–139.89)	105.28 ± 1.63 (81.78–132.00)	.27
Creatinine (µmol/L)	81.27 ± 1.75 (66.00–99.00), EHT only: *n* = 26	81.71 ± 3.32 (64.00–103.00), EHT only: *n* = 14	.90
Sodium (mmol/L)	140.55 ± 0.36 (138.00–144.00), EHT only: *n* = 22	141.00 ± 0.59 (137.00–145.00), EHT only: *n* = 13	.49
Calcium (mmol/L)	2.38 ± 0.02 (2.11–2.58), EHT only: *n* = 22	2.38 ± 0.03 (2.22–2.56), EHT only: *n* = 13	.94
Potassium (mmol/L)	4.06 ± 0.03 (3.70–4.30), EHT only: *n* = 22	4.13 ± 0.07 (3.90–4.70), EHT only: *n* = 13	.27

	participants who completed the follow-up, *n* = 97	participants who dropped out, *n* = 47	*p*
Inflammation			
IL-6 (pg/mL)	0.61 ± 0.09 (0.03–8.30), *n* = 96	0.55 ± 0.05 (0.09–1.48), *n* = 45	.66
TNF-α (pg/mL)	2.04 ± 0.07 (0.80–4.91), *n* = 96	1.82 ± 0.09 (0.70–3.68), *n* = 45	**.059**
CRP (µg/mL)	2.03 ± 0.21 (0.07–9.59), *n* = 88	2.60 ± 0.21 (0.35–7.59), *n* = 41	.10
Blood lipids			
tChol (nmol/l)	4.88 ± 0.11 (2.71–7.89), *n* = 96	5.04 ± 0.18 (2.80–7.79), *n* = 44	.43
LDL (nmol/l)	3.12 ± 0.10 (1.13–5.87), *n* = 96	3.25 ± 0.16 (1.52–6.53), *n* = 44	.46
HDL (nmol/l)	1.44 ± 0.03 (0.91–2.21), *n* = 96	1.44 ± 0.05 (0.87–2.40), *n* = 44	.89
Aldosterone			
Awakening (pg/ml)	47.81 ± 1.60 (9.19–89.64)	50.79 ± 3.41 (14.69–143.93)	.72
15 min (pg/ml)	49.64 ± 1.87 (6.82–115.55)	53.72 ± 5.02 (13.58–258.11)	.63
30 min (pg/ml)	51.87 ± 2.11 (6.04–127.35)	52.22 ± 2.54 (6.82–115.55)	.76
45 min (pg/ml)	55.19 ± 2.54 (6.04–164.48)	56.28 ± 3.07 (19.66–105.57)	.57
60 min (pg/ml)	54.39 ± 2.34 (4.97–188.77)	52.40 ± 3.06 (14.22–103.28)	.80

Notes. Values are *M* ± *SEM*; CHD = coronary heart disease patients; EHT = essential hypertensive individuals; NT = normotensive individuals; BMI = body mass index; DBP = diastolic blood pressure; SBP = systolic blood pressure; MAP = mean arterial blood pressure.

Supplementary Table 3

Medication intake at follow up

Medication	CHD (*n* = 42)	EHT (*n* = 26)	NT (*n* = 29)
RAAS-modulating medication			
At least one of the following medications: ACE inhibitors, AT-II receptor antagonists, aldosterone antagonists	26	4	
At least one of the following medications: ACE inhibitors, AT-II receptor antagonists, aldosterone antagonists, beta blockers	32	4	
All medication			
ACE inhibitors	12	2	
AT-II receptor antagonists	15	2	
Aldosterone antagonists	1		
Alpha-1 blockers		1	
Alpha and beta blockers			
Beta blockers	21		
Calcium channel blocker	11	1	
Antiarrhythmics	2		
Anticoagulants	4	1	1
Antiplatelets	29	1	1
Lipid lowering drugs	29	3	
Diuretics	6	1	
Antidiabetics	1	1	
Insulin	1		1
Antiepileptics	1		
Antiparkinsonian drugs	2		

Medication	CHD (n = 42)	EHT (n = 26)	NT (n = 29)
Antidepressants	1	1	
Others	12	3	2

Note. CHD = coronary heart disease patients; EHT = essential hypertensive individuals; NT = normotensive individuals.

Supplementary Figure 1

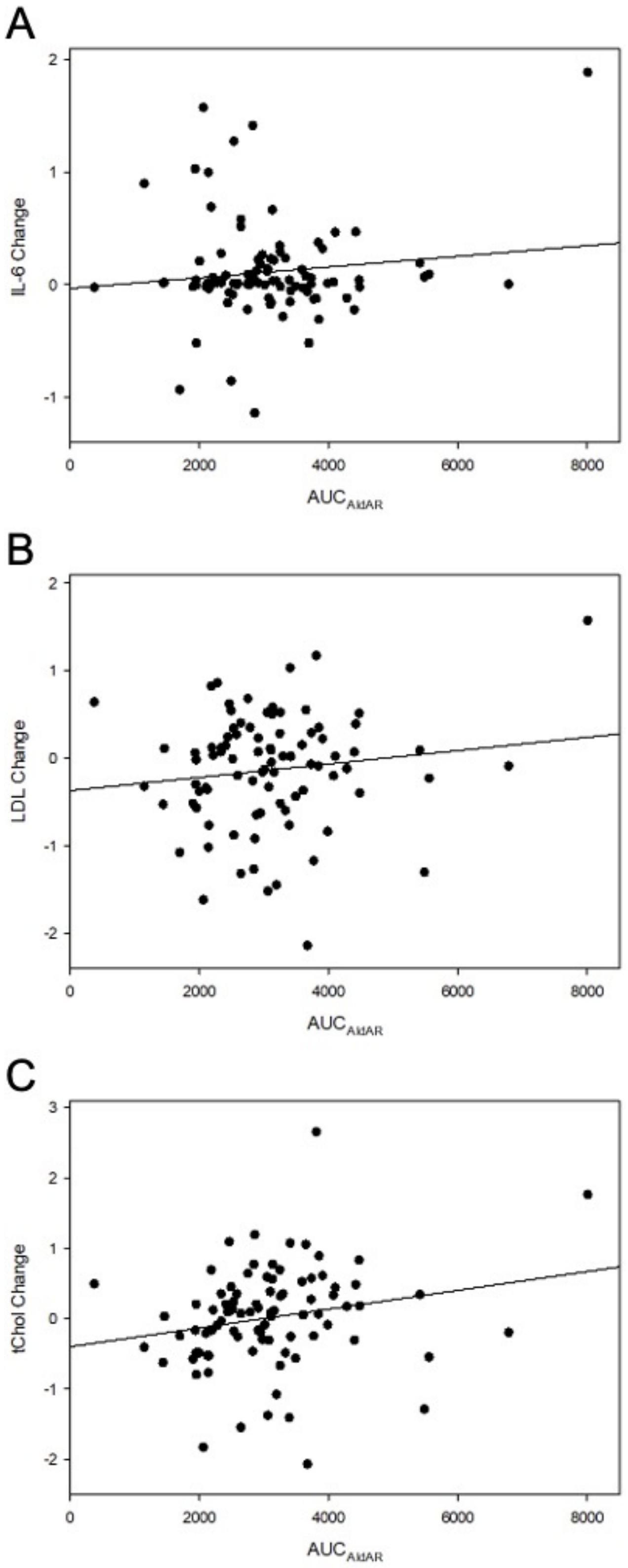

Supplementary Figure 1. Plots depict linear associations of AUC_{AldAR} with changes (follow-up minus baseline assessments) in A: IL-6 (n = 80), B: LDL (n = 80), and C: tChol (n = 87).
